# Detection of *Mycobacterium ulcerans* DNA in the Environment, Ivory Coast

**DOI:** 10.1371/journal.pone.0151567

**Published:** 2016-03-16

**Authors:** Roger Bi Diangoné Tian, Sébastian Niamké, Hervé Tissot-Dupont, Michel Drancourt

**Affiliations:** 1 Aix Marseille Université, URMITE, UMR, CNRS 7278, IRD 198, INSERM 1095, Faculté de Médecine, Marseille, France; 2 Laboratoire de biotechnologies, UFR Biosciences, Université Félix Houphouet Boigny Abidjan, Côte d’Ivoire; Naval Research Laboratory, UNITED STATES

## Abstract

**Background:**

Ivory Coast is a West African country with the highest reported cases of Buruli ulcer, a disabling subcutaneous infection due to *Mycobacterium ulcerans*. However, the prevalence of environmental *M*. *ulcerans* is poorly known in this country.

**Methods:**

We collected 496 environmental specimens consisting of soil (n = 100), stagnant water (n = 200), plants (n = 100) and animal feces (n = 96) in Ivory Coast over five months in the dry and wet seasons in regions which are free of Buruli ulcer (control group A; 250 specimens) and in regions where the Buruli ulcer is endemic (group B; 246 specimens). After appropriate total DNA extraction incorporating an internal control, the *M*. *ulcerans* IS*2404* and KR-B gene were amplified by real-time PCR in samples. In parallel, a calibration curve was done for *M*. *ulcerans* Agy99 IS*2404* and KR-B gene.

**Results:**

Of 460 samples free of PCR inhibition, a positive real-time PCR detection of insertion sequence IS*2404* and KR-B gene was observed in 1/230 specimens in control group A versus 9/230 specimens in group B (*P* = 0.02; Fisher exact test). Positive specimens comprised seven stagnant water specimens, two feces specimens confirmed to be of *Thryonomys swinderianus* (agouti) origin by real-time PCR of the *cyt*b gene; and one soil specimen. Extrapolation from the calibration curves indicated low inoculums ranging from 1 to 10^2^ mycobacteria/mL.

**Conclusion:**

This study confirms the presence of *M*. *ulcerans* in the watery environment surrounding patients with Buruli ulcer in Ivory Coast. It suggests that the agouti, which is in close contacts with populations, could play a role in the environmental cycle of *M*. *ulcerans*, as previously suggested for the closely related possums in Australia.

## Introduction

Ivory Coast is the country most affected by Buruli ulcer, a WHO-reportable disabling subcutaneous infection caused by to *Mycobacterium ulcerans* [1]. Indeed, more than 1,000 cases are reported every year in this country, despite the efforts of public health units [1]. The reservoir of *M*. *ulcerans* is unknown but the absence of evidence of human-to-human transmission suggests an environmental reservoir. Accordingly, several epidemiological publications in West Africa have pointed to contact with stagnant water as a significant risk factor for contracting Buruli ulcer [2–5]. The slow growth of *M*. *ulcerans* makes it easily overgrown by contaminant microorganisms; therefore, the search for this pathogen in the environment has been mainly based on the molecular detection of *M*. *ulcerans* DNA, i.e. chromosomal and plasmidic sequence insertion IS*2404* and the KR-B plasmidic gene encoding a polyketide synthase participating into the biosynthesis of the mycolactone toxin [6,7].

In Ivory Coast, the use of these molecular methods confirmed the detection of *M*. *ulcerans* in freshwater insects [7–10]. Two isolates, Nau CI 001 and Nau CI 002, have also been cultivated from *Naucoris* spp. water insects [8] but this observation has been disputed [11,12]. So far, only one environmental isolate has been firmly sequence-confirmed from an aquatic Hemiptera (in Benin, another West African country) [11].

In this study, we launched a new environmental sampling campaign, investigating soil, stagnant and running water, plants and feces of an endemic small mammal, *Thryonomys swinderianus* (agouti), in four regions of Ivory Coast. The aim was to develop understanding about the ecological and geographical distribution of the pathogen in this country for Buruli ulcer.

## Materials and Methods

### Environmental samples

**Fig 1:**
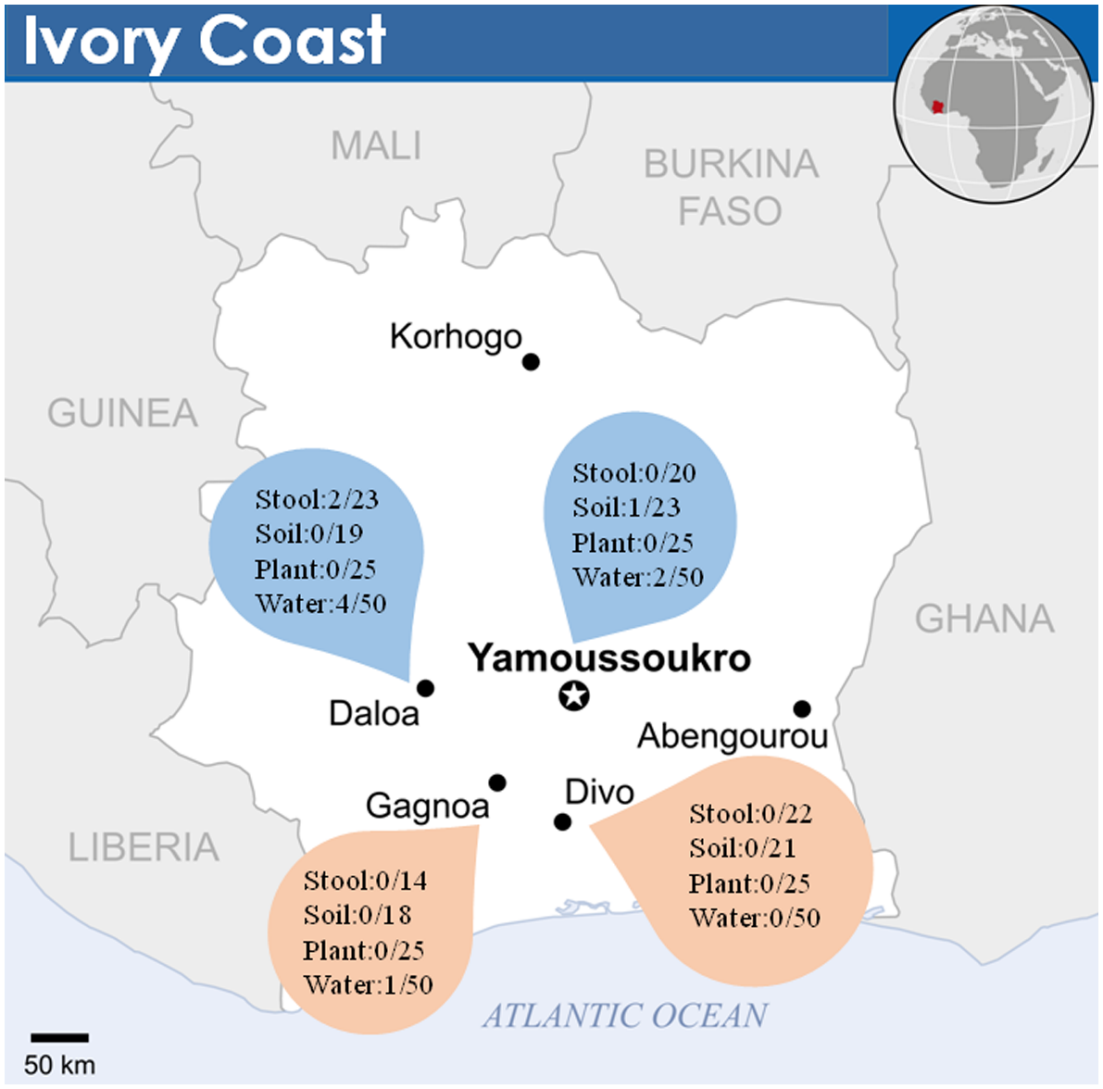
Map of Ivory Coast (Based on OCHA/ReliefWeb) showing the Buruli ulcer endemic areas based on World Health Organization data (2013) and location of studied environmental samples. Each circle figures the number of positive samples over collected samples. Blue teardrops, endemic areas; tan teardrops, non-endemic areas.

We carried-out a campaign of environmental samplings between June and October 2014 (June-July is the wet season and August-October is the dry season) in four regions of Ivory Coast comprising regions of low incidence of Buruli ulcer (control group A) and regions of high incidence of Buruli ulcer (group B) as documented by official records of active surveillance by the “Programme National de Lutte contre l’Ulcer de Buruli” (National Program against Buruli Ulcer) [1] and confirmed to one of us (Tian B.D.R.) by interviewing nurses and village leaders (Fig 1). This work was carried out with the approval of the national program against Buruli ulcer in Ivory Coast (PNLCI) and no further permissions were required for the environmental sampling of common goods. This study did not involve any endangered or protected species, and no vertebrates.

A series of water samples (n = 200) were collected in endemic areas (n = 100) and in non-endemic areas (n = 100) at the edge and middle of stagnant waters at an average 50-cm depth. A 10 mL volume of water was collected in a 15 mL sterile tube (Sarstedt, Nümbrecht, Germany) which was immediately closed, sealed and placed in a cooler at 4°C. Non-identified plants were collected mainly in permanent and temporary stagnant waters and rivers in endemic areas (n = 50) and in non-endemic areas (n = 50). One hundred plant fragments from endemic plants in Ivory Coast including roots, stems and leaves were harvested and each sample placed into a sterile 15 mL Falcon tube stored as described above. Soil samples (n = 100; endemic areas, n = 50; non-endemic areas n = 50) were collected at half a meter or a meter from the water (river, standing water). Finally, feces fragments collected from the ground (n = 96; endemic areas, n = 48; non-endemic areas, n = 48), morphologically identified by a traditional hunter as *Thryonomys swinderianus* (agouti) stools, were put into a sterile tube as described above. Molecular confirmation of *T*. *swinderianus* feces was done by cytochrome b gene standard PCR. All feces specimens were confirmed to be *T*. *swinderianus* (agouti) by exhibiting a *cyt*b amplification with Ct values of 25. All samples were stored at 4°C in our laboratory for analysis. Collected plant fragments and fecal samples did not come from species listed as endangered.

### DNA extraction

Total soil DNA was extracted using a soil DNA extraction kit according to the manufacturer’s instructions (NucleoSpin^®^Soil, Macherey-Nagel, Düren, Germany). Total stool DNA was extracted using a stool extraction kit according to the manufacturer’s instructions (QIAmp^®^, DNA Stool, Qiagen, Stochach, Germany). Total DNA was extracted from plants and sedimented water using the NucleoSpin Tissue Kit (Macherey—Nagel). *M*. *ulcerans* Agy99 DNA was extracted using a commercial kit (Tissue Kit, Macherey-Nagel) as a positive control. PCR inhibition was assessed by adding 10μL of internal control into 190μL of sample, as previously described [13]. Further, efficiency of DNA extraction for *M*. *ulcerans* in feces was assessed by spiking a final inoculum of 10^6^ *M*. *ulcerans* Agy99 colony-forming units (cfu)/mL agouti feces. These artificially infected feces were extracted as described above. In each extraction batch of 25 samples, two negative controls consisting of distilled water were included. All stool or soil specimens demonstrating PCR inhibition were diluted 1:10 and 1:100 in PBS before being tested again.

### Real-time PCR amplifications

In the first step, a calibration curve was made for each one of the two *M*. *ulcerans* DNA targets by using real-time PCR as described below, in a series of 10-fold *M*. *ulcerans* Agy99 suspensions from 10^6^ cfu/mL to 1 cfu/mL; suspensions were extracted as described above in triplicate. Then, for each specimen, IS*2404* and KR-B were tentatively amplified using real-time PCR (RT-PCR) performed incorporating RT-PCR reagents (Takyon, Eurogentec, Liege, Belgium) and primers and probes as previously described [6], in a CFX 96^™^ real time PCR thermocycler and detection system (BIO-Rad, Marnes-la-Coquette, France). PCR was conducted under a 20 μL volume containing 5 μL of DNA, 0.5 μL of each primer, 0.5 μL of probe, 3.5 μL of sterile water and 10 μL of mastermix (Eurogentec). The RT-PCR program comprised one cycle at 50°C for two minutes and 40 cycles at 95°C for 15 seconds and 60°C for one minute. Two negative controls for 25 samples were incorporated into each PCR run. All samples were tested in duplicate. A specimen was considered as positive for the detection of *M*. *ulcerans* when both the insertion sequences IS*2404* and the KR-B gene detection was positive (Ct ≤ 40 cycles) in replicates. The Ct value cut-off was chosen in order to increase the sensitivity of the detection.

### Statistical analysis

According to the low effectives, frequencies of quantitative data were compared using the Fisher’s exact test. A difference was considered significant when the *P* value < 0.05.

## Results

Between June and October 2004, a total of 496 environmental specimens were collected in Ivory Coast, consisting of 100 soil specimens, 200 stagnant water specimens, 100 plant specimens and 96 feces specimens. A total of 246 specimens were collected in control group A negative control geographic areas and a total of 250 specimens were collected in Buruli ulcer endemic geographic group B areas.

In preliminary RT-PCR experiments, the negative controls remained negative and the *M*. *ulcerans* positive control yielded consistent calibration curves for IS*2404* and KR-B gene (S1 Table). As for the samples, a total of 36/496 (7.2%) samples including 17 stool samples and 19 soil samples did not yield amplification of the internal control, suggesting PCR inhibition. None of these 36 samples (pure and diluted 1:10 and 1:100) yielded any amplification for *M*. *ulcerans* and they were deleted from further analysis. Of 460 PCR inhibition-free samples, 43 (9.3%) yielded at least one positive RT-PCR detection of IS*2404* or KR (Table 1). Of these 43 specimens, only 10/460 (2.1%) specimens were positive for IS*2404* and KR and fulfilled our definition of a PCR- positive specimen. Positive samples yielded *M*. *ulcerans* inoculums estimated to be of 1 cfu/mL to 10^2^ cfu/mL. The prevalence of the positive detection of IS*2404* was significantly higher in endemic group B geographic areas (19/230, 8.3%) than in negative control group A geographic areas (4/230, 1.7%)(P = 0.002; OR = 5.1 [1.7–15.2]. The prevalence of the positive detection of *M*. *ulcerans* was significantly higher in endemic group B geographic areas (9/230, 3.9%) than in negative control group A geographic areas (1/230, 0.04%) (*P* = 0.02, Fisher’s exact test; OR = 9.33 [1.17–74.22]. These 10 *M*. *ulcerans*-positive specimens consisted of seven water specimens collected from permanent puddles in the Haut-Sassandra region, the Yamoussoukro district and the Gôh region in June-October, two *T*. *swinderianus* (agouti) feces specimens collected in the Haut-Sassandra region in October and one soil specimen collected in Yamoussoukro district in October. Altogether, 6/281(2.5%) specimens collected in the dry season were positive versus 4/215 (1.9%) specimens collected in the wet season (*P* = 0.76).

**Table 1:** Molecular detection of *M*. *ulcerans* in environment, Ivory Coast and number of environmental samples free of PCR inhibition and positive for the RT-PCR detection of *M*. *ulcerans* (CFU, colony-forming units, Ct cycle threshold).

	IS*2404*			KR-B			IS*2404* and KR-B True positive	Estimated CFU/mL
	positive	Ct	CFU/mL	positive	Ct	CFU/mL		
Soil (n = 81)	2	33	10	1	35	10	1	10
		37	10					
Plant (n = 100)	0	0	0	0	0	0	0	0
Stool (n = 79)	5	33	10	2	35	10^2^	2	10^2^
		37	10		38	10		10
		35	10		-			
		38	10^0^		-			
		34	10		-			
		39	10		-			
Water (n = 200)	16	36	10	7	38	10	7	10
		31	10		33	10^2^		10^2^
		30	10^2^		32	10^2^		10^2^
		33	10		35	10		10
		32	10		34	10		10
		35	10		36	10		10
		31	10		33	10^2^		10^2^
		38	10^0^		-			
		39	10^0^		-			
		35	10		-			
		36	10		-			
		37	10		-			
		39	10^0^		-			
		36	10		-			
		38	10^0^		-			
		37	10		-			
Total (n = 460)	23			10			10	

## Discussion

Ivory Coast is the country most affected by Buruli ulcer and many efforts have been undertaken to clarify risk factors, significantly linking cases with proximity to dams used for irrigation and aquaculture [2]. However, surprisingly, few data have been reported from the direct search for the causative agent *M*. *ulcerans* in these environments in Ivory Coast. Here we report the results of the first systematic investigation in this West African country. Based on data reported in similar investigations conducted in Benin [5], Ghana [14] and Australia [15], we looked for *M*. *ulcerans* DNA in soil, stagnant water, aquatic plants and feces of the most prevalent endemic mammal in Ivory coast, *T*. *swinderianus*. We voluntarily neglected the investigation of water insects as they have been extensively studied in Ivory Coast and have previously shown to be infected by *M*. *ulcerans* [7–10]. In order to ensure the specificity of RT-PCR detection, we retained as positive only those specimens exhibiting a positive amplification for one insertion sequence IS*2404* plus the KR-B gene, in the presence of negative controls according to standard interpretation. Indeed, none of these three markers is specific by itself for *M*. *ulcerans*, since IS*2404* has been also detected in *Mycobacterium liflandii* [16], and IS*2404* and KR-B gene in some isolates of *Mycobacterium liflandii* [17], of *Mycobacterium marinum* [18] and *Mycobacterium pseudoshottsii* [19].

Using these stringent parameters, we detected *M*. *ulcerans* DNA in only 2.1% environmental specimens. Apart from one positive stagnant water specimen in one non-endemic region, it is significant to note that all positive specimens were collected in areas endemic for Buruli ulcer. This observation agrees with the current knowledge that *M*. *ulcerans* is an environmental organism of as yet uncertain sources [4–7]. Alternatively, this difference could be due to the difference in the composition of sources between the endemic and non-endemic areas here investigated. Interestingly, 7/10 (70%) positive specimens had been collected during the dry season [3, 20–22]. The detection of *M*. *ulcerans* in seven stagnant water specimens was not surprising, as similar observations have been done in several studies in neighboring African countries [5] as well as in Australia [14] and South America [20]. We detected *M*. *ulcerans* inocula 100 times much lower than those previously reported in Benin; however, different methodology between the two studies may account for this difference [5]. The proximity to dams and stagnant water resulting from irrigation has been demonstrated as a risk factor for Buruli ulcer in Ivory Coast [2] and other countries [5, 15, 20]. The role of water however remains unknown as it could be a reservoir by itself or could support a reservoir for *M*. *ulcerans* but does not resume the cycle of this organism which is presumably inoculated to cause Buruli ulcer [5, 23–25]. More unexpected was the observation of two soil specimens collected during the dry season at 50 cm and one meter from stagnant water. Soil has rarely been found positive for *M*. *ulcerans*, except in Benin [5]. In fact, we recently confirmed this field observation, by observing a four-month survival of *M*. *ulcerans* in experimentally contaminated soil [26].

The detection of *M*. *ulcerans* in two feces specimens collected 21 days apart in two different villages (Daloa, Haut-Sassandra Region) from *T*. *swinderianus* (agouti) mammals was also unexpected. This observation is very interesting as the agouti is known to be endemic in watery areas resulting from irrigation efforts in Ivory Coast where it destroys rice fields and is in close contacts with populations as it is hunted for consumption of its meat. Moreover, *T*. *swinderianus* was shown to develop Buruli ulcer after experimental subcutaneous inoculation of *M*. *ulcerans* [27]. This observation recalls the detection of *M*. *ulcerans* in the feces of possums in Australia in geographic regions which are endemic for Buruli ulcer [28]. Both animals are small mammals. Combined with earlier observations in Ivory Coast [12], the observations reported here support a natural cycle of *M*. *ulcerans* which is more complex than previously anticipated. In Ivory Coast, additional field and experimental studies will need to be conducted in order to interpret our observations and to determine whether the digestive tract of the agouti is merely contaminated by bypassing *M*. *ulcerans* organisms from contaminated water; or if the agouti plays any role in the environmental cycle of *M*. *ulcerans*, through fecal contamination of soil and direct contamination of populations.

## Supporting Information

S1 Table Calibration curve of IS*2404* and KR-B gene obtained with *M*. *ulcerans* Agy99. (DOCX)
