# Supplementary material for: Detection of Mycobacterium ulcerans DNA in the Environment, Ivory Coast
Source: PLoS One. 2016 Mar 16;11(3):e0151567. doi: 10.1371/journal.pone.0151567 (PMC4794205; doi:10.1371/journal.pone.0151567)
Supplement: S1 Table — (DOCX) [file pone.0151567.s001.docx]

| *Mycobacterium ulcerans* Agy99 (CFU/mL) | Ct (IS*2404*) | Ct (KR-B) |
| --- | --- | --- |
| 10^7^ | 9.55 ± 0.06 | 14.36 ± 0.04 |
| 10^6^ | 13.60 ± 0.11 | 17.88 ± 0.12 |
| 10^5^ | 17.09 ± 0.08 | 20.98 ± 0.11 |
| 10^4^ | 21.21 ± 0.09 | 25.22 ± 0.02 |
| 10^3^ | 25.23 ± 0,39 | 29.30 ± 0.06 |
| 10^2^ | 28.53 ± 0.14 | 32.75 ± 0.21 |
| 10^1^ | 31.70 ± 0.12 | 36.35 ± 1.10 |
| 10^0^ | 38.31 ± 1.09 | 0 ± 0 |

Supplementary Table 1.
